# Regionally-triggered geomagnetic reversals

**DOI:** 10.1038/s41598-024-59849-z

**Published:** 2024-04-26

**Authors:** Filipe Terra-Nova, Hagay Amit

**Affiliations:** Nantes Université, Univ Angers, Le Mans Université, CNRS, Laboratoire de Planétologie et Géosciences, LPG UMR 6112, 44000 Nantes, France

**Keywords:** Geodynamics, Geomagnetism, Core processes

## Abstract

Systematic studies of numerical dynamo simulations reveal that the transition from dipole-dominated non-reversing fields to models that exhibit reversals occurs when inertial effects become strong enough. However, the inertial force is expected to play a secondary role in the force balance in Earth’s outer core. Here we show that reversals in numerical dynamo models with heterogeneous outer boundary heat flux inferred from lower mantle seismic anomalies appear when the amplitude of heat flux heterogeneity is increased. The reversals are triggered at regions of large heat flux in which strong small-scale inertial forces are produced, while elsewhere inertial forces are substantially smaller. When the amplitude of heat flux heterogeneity is further increased so that in some regions sub-adiabatic conditions are reached, regional skin effects suppress small-scale magnetic fields and the tendency to reverse decreases. Our results reconcile the need for inertia for reversals with the theoretical expectation that the inertial force remains secondary in the force balance. Moreover, our results highlight a non-trivial non-monotonic behavior of the geodynamo in response to changes in the amplitude of the core-mantle boundary heat flux heterogeneity.

## Introduction

In order to simulate magnetic field reversals using self-consistent 3D simulations of core convection in a spherical shell^1,2^, the convection should be sufficiently vigorous^3^. However, under such conditions inertial effects become significant^4–6^ hence the fluid dynamics might depart from an Earth-like force balance. Earth’s core dynamics are governed by a zeroth-order quasi-geostrophic balance, with the ageostrophic Coriolis force expected to be balanced by Archimedes and Lorentz forces (termed QG-MAC, see^7–12^). It therefore remains a challenge to simulate reversals while maintaining an Earth-like force balance with secondary inertial effects.

The above mentioned systematic parametric studies of numerical dynamos were performed using homogeneous boundary conditions. However, heterogeneous boundary conditions have often been invoked in numerical dynamos to recover various observations related to the geomagnetic field, its secular variation and the core convection that generates it (e.g.^13–23^). Over much longer timescales, mantle control on the geodynamo was also proposed to explain the strongly time-dependent paleomagnetic reversal frequency (e.g.^24–27^).

Here we show that the impact of lower mantle heterogeneity on the geodynamo may reconcile the existence of reversals and an Earth-like force balance. We start from a dynamo model with homogeneous boundary conditions in the well-established, dipole-dominated non-reversing regime. Then we impose a heterogeneous outer boundary heat flux inferred from a lower mantle tomography model (Fig. SM1;^28^) and we gradually increase the amplitude of the heat flux heterogeneity $q^*$ (see Methods and Table SM1).

## Results

**Figure 1:**
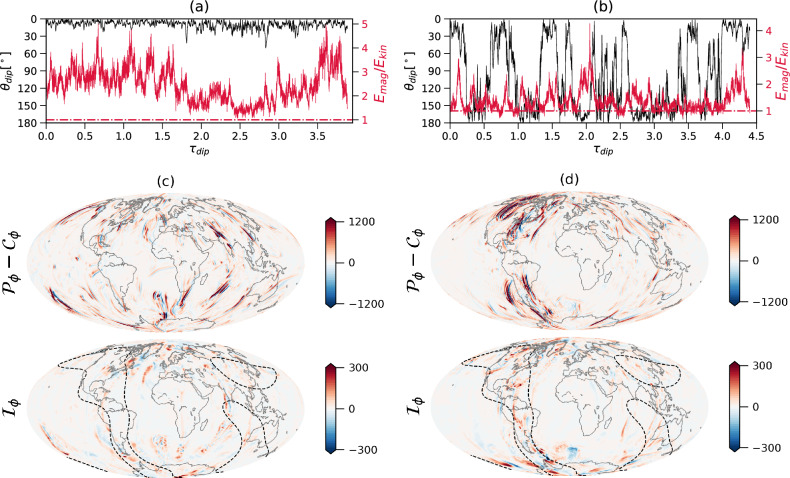
The distance of the dipole axis from the north geographic pole (black) and the ratio between magnetic energy and kinetic energy (red) vs. time in units of dipole diffusion times for two numerical dynamo simulations. In both cases the Ekman number is $E=1\times 10^{-4}$, the Rayleigh number is $Ra=4\times 10^7$, the Prandtl number is $Pr=1$ and the magnetic Prandtl number is $Pm=8$ (for definitions of the non-dimensional numbers see Methods). The amplitude of the imposed outer boundary heat flux heterogeneity (see Fig. SM1) is $q^*=0.4$ (**a**) and $q^*=1.0$ (**b**). Red point-dashed lines denote $E_{mag}/E_{kin} = 1.0$. The non-dimensional azimuthal components of the ageostrophic Coriolis and Inertia forces $\mathcal {P}_\phi -\mathcal {C}_\phi$ and $\mathcal {I}_\phi$ at the top of the shell (radial level $r/r_o$ = 0.95 where $r_o$ is the core radius) for a typical snapshot of the model with $q^*=0.4$ are shown in (**c**), and the same forces for the model with $q^*=1.0$ are shown in (**d**). Note the different scales of the energy ratios in (**a**) vs. (**b**) and the different scales in ageostrophic Coriolis vs. inertial forces in (**c**,**d**). Dashed black contours denote half maximum of the imposed outer boundary heat flux anomaly in (**c**,**d**).

Figure 1 compares two dynamo models, one with a relatively low amplitude of heat flux heterogeneity (Figs. 1a,c) vs. another with a larger $q^*$ value (Figs. 1b,d). In the case of a relatively weak boundary heterogeneity, the ratio of magnetic to kinetic energies in the shell [which is considered as a proxy to reversals, see^11^] is larger than unity and the field never reverses (Fig. 1a). At the top of the shell, the inertial force is significantly smaller than the dominant first order ageostrophic Coriolis force (Fig. 1c), as expected for a QG-MAC force balance^8^. In contrast, in the case of a stronger boundary heterogeneity, the ratio of magnetic to kinetic energies in the shell is lower including some periods in which it dips below unity, and the field exhibits reversals (Fig. 1b). Overall the inertial force is still significantly weaker than the ageostrophic Coriolis force - note that the peak of the former is four times smaller than the peak of the latter (see scale difference in Fig. 1d). In our dynamo models the ageostrophic Coriolis force in the two horizontal directions is indeed mostly balanced by the Lorentz force (see Fig. SM2). However, some localized inertial force signatures appear, especially below regions of large outer boundary heat flux (see dashed contours in Fig. 1d). In this snapshot strong localized inertial force features are concentrated mostly below the Americas, though at other snapshots these structures alternate between the Americas and east Asia, the two regions where the imposed outer boundary heat flux is large. Away from these narrow stripes, the inertial force is much weaker. Volumetric averages of the forces in our dynamo models (Fig. SM3) confirm the dominance of quasi-geostrophy, with the residual ageostrophic Coriolis force balanced by the buoyancy force at large scales and the Lorentz force at small scales (i.e. QG-MAC,^11^). The inertial force is an order of magnitude smaller than the ageostrophic Coriolis force (Fig. SM3).

**Figure 2:**
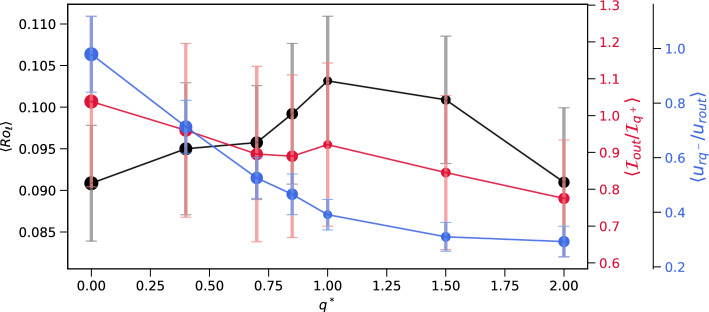
Diagnostics of the dynamo models vs. the outer boundary heat flux anomaly amplitude $q^*$. The local Rossby number $Ro_\ell$ (13) is in black. The size of the symbols is proportional to the dipolarity $f_{dip}$ (15). The ratio between the RMS azimuthal component of the inertial force at $r/r_o=0.95$ outside/inside regions where $\delta q > \frac{1}{2} \delta q_{max}$ (17–18) is in red. The ratio between the RMS radial velocity at $r/r_o=0.95$ inside/outside regions where $\delta q < \frac{1}{2} \delta q_{min}$ (19–20) is in blue. Error bars denote temporal variability.

Figure 2 summarizes some key diagnostics for all the dynamo models, which reveal the dependence of the reversibility on the amplitude of heat flux heterogeneity $q^*$. Increasing effects of mantle control from the homogeneous case up to $q^*=1.0$ increases the tendency of the dynamo models to reverse, as evident by the increase in the local Rossby number (black) and decrease in the dipolarity of the field (symbol size) (Fig. 2) that result in the increase in the time-average dipole tilt (Fig. SM4), in agreement with most previous studies (e.g.^6,25^). However, further increase in $q^*$ causes, somewhat surprisingly, opposite trends in these diagnostics, i.e. a decrease in the reversibility (Figs. 2 and SM4).

**Figure 3:**
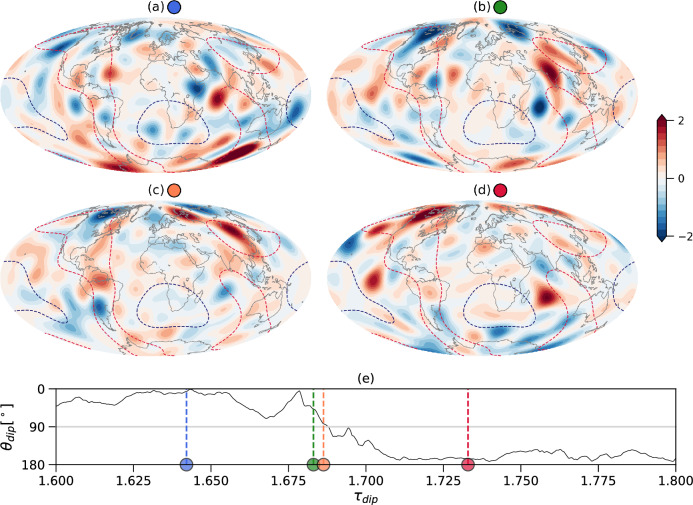
Snapshots of a simulated reversal. Non-dimensional radial magnetic field on the outer boundary of the dynamo model with $q^*=1.5$ truncated at spherical harmonic degree and order 14. Dashed red and blue contours denote half maximum and half minimum of the imposed outer boundary heat flux anomaly respectively. The maps show the radial field before (**a**), during (**b**,**c**) and after (**d**) the reversal. The distance of the dipole axis from the north geographic pole vs. time in units of dipole diffusion times with the corresponding times for the four snapshots denoted by dashed vertical lines is given in (**e**).

These two opposite trends can be explained in terms of our regional measures of mantle control (see Methods). For moderate mantle control ($q^*<1$), increasing $q^*$ leads to stronger inertial force at regions of large outer boundary heat flux (red dashed contours in Fig. SM1, red in Fig. 2), i.e. the reversals are triggered regionally by inertial effects, while globally inertia is far too weak to play a role in the first order force balance. The impact of the core-mantle boundary (CMB) heat flux heterogeneity is also visible in the morphology of the radial magnetic field on the outer boundary during a reversal (Fig. 3). Before (Fig. 3a) and after (Fig. 3d) the reversal, intense high-latitude normal polarity flux patches (typically two at each hemisphere) that maintain the axial dipole are preferentially located below regions of high CMB heat flux (dashed red contours in Fig. 3). This configuration is disrupted during the reversal (see movie in SM); however, the magnetic field structures that dominate the transitional field are still concentrated in the high CMB heat flux longitudinal stripes below the Americas and east Asia. In Fig. 3b large positive mid-latitude magnetic flux patches below east Asia weaken the axial dipole, while in Fig. 3c small-scale magnetic flux patches below the Americas dominate the western hemisphere while the southern high latitudes are devoid of intense magnetic flux.

**Figure 4:**
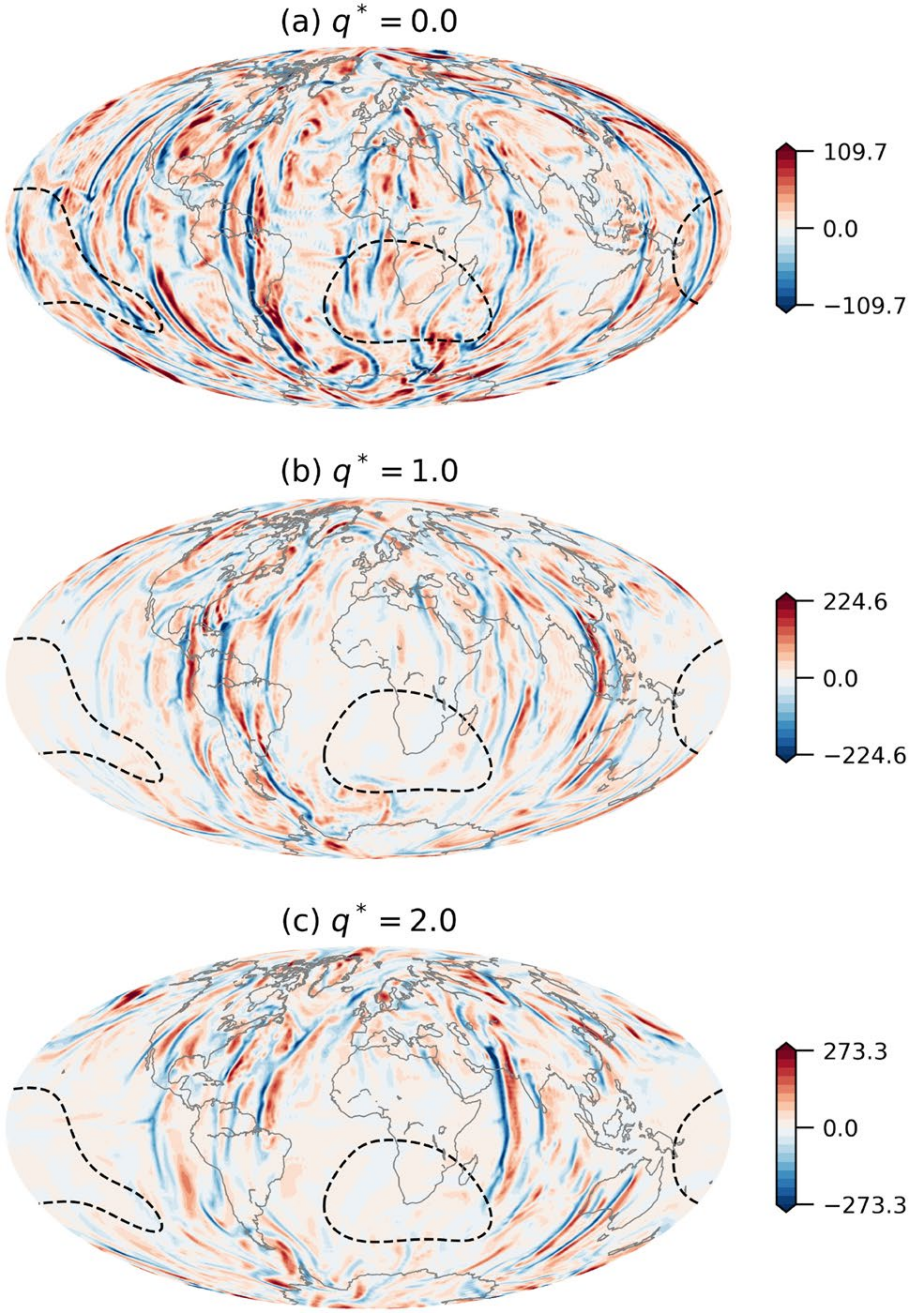
Non-dimensional radial velocity at the top of the shell ($r/r_o=0.95$) for dynamo models with (**a**) $q^*=0.0$, (**b**) $q^*=1.0$ and (**c**) $q^*=2.0$. Dashed contours denotes the regions where $\delta q < \frac{1}{2} \delta q_{min}$ (see Fig. SM1). Note the different scales.

For stronger mantle control ($q^*>1$), the effect of increasing $q^*$ is related to other CMB features. In these dynamo models, increasing $q^*$ leads to more extensive regional subadiabatic conditions^19,20^ below Large Low Shear wave Velocity Provinces (LLSVPs; blue dashed contours in Fig. SM1, blue in Fig. 2). In these regions convection is suppressed and skin effects diffuse effectively small-scale magnetic fields^29^ hence the dipole becomes relatively stronger and the likelihood for a reversal decreases. The distribution of radial velocity at the top of the shell (Fig. 4) illustrates the emergence of regional subadiabatic conditions when the amplitude of the outer boundary heat flux heterogeneity exceeds unity. In the homogeneous case (Fig. 4a) convective cells extend until the outer boundary sporadically over the entire globe. When $q^*=1$ (Fig. 4b) these convective structures are nearly absent from the regions below LLSVPs (see dashed contours), and even less radial flows reach the outer boundary in these regions for the dynamo model with $q^*=2$ (Fig. 4c).

## Discussion

We showed that an increase in the amplitude of the outer boundary heat flux heterogeneity within moderate values of $q^*<1$ leads to a transition from non-reversing to reversing dynamos (Figs. 2 and SM4). In our dynamo models, reversals of the geomagnetic field are triggered below the Americas and east Asia where the CMB heat flux is anomalously large. Below these regions, relatively more heat is extracted from the core^30^, leading to stronger convection and inertial effects (Fig. 1). Sahoo and Sreenivasan^22^ argued that enhanced turbulent conditions below regions of stronger CMB heat flux lead to fragmentation of the magnetic field structures. We propose that this regional fragmentation may eventually result in a global polarity reversal. While these regional inertial effects are strong enough to produce reversals, away from these large outer boundary heat flux regions inertial effects are secondary to the dominant first order ageostrophic Coriolis force, as expected for Earth’s outer core^7,11^. Upon further increase in the amplitude of outer boundary heat flux heterogeneity, the trend somewhat surprisingly changes, and the reversibility decreases (Figs. 2 and SM4). At $q^*>1$ the heat flux becomes regionally subadiabatic below the African and Pacific LLSVPs^19,20^ and the convection there vanishes (Fig. 4), leading to skin effects that suppress small-scale contributions to the geomagnetic field^29^ hence stabilize the dipole.

Somewhat various ranges were considered for the dipolarity $f_{dip}$ that characterizes dipole-dominated dynamos (see^31^, and references therein). In addition, various values were reported for the critical local Rossby number that was proposed to mark the transition from dipole-dominated non-reversing (characterized by low $Ro_\ell$) to multipolar reversing dynamos (characterized by large $Ro_\ell$). This critical local Rossby number may depend on the convection style^4,6,32^. In addition, the transition might not be sharp, i.e. some overlaps between these two dynamo regimes may prevail, which are of particular interest for recovering the behavior of the geomagnetic field over distinctive timescales. Menu et al.^33^ reported non-reversing dynamos with large $Ro_\ell$. Our dynamo models highlight the impact of the boundary heterogeneity on the critical $Ro_\ell$. Given a large enough amplitude of outer boundary heat flux heterogeneity, we found reversing dynamos with a relatively low $Ro_\ell$ of less than 0.1 (Fig. 2).

Our results of increasing reversibility for increasing $q^*$ are in agreement with most previous studies (e.g.^6,25^). In contrast, Mound and Davies^34^ found that a reversing dynamo with homogeneous boundary conditions becomes non-reversing when $q^*$ is increased. However, they introduced large leaps in $q^*$, possibly skipping the moderate $q^*$ regime in which reversibility increases with $q^*$ and reaching directly from homogeneous to the large $q^*$ regime in which reversibility decreases with $q^*$ (Fig. 2).

Stratification at the top of Earth’s core may have emerged by various mechanisms, e.g. primordial origin^35^, compositional convection^36^, and more. Our finding that the reversibility decreases with $q^*$ when it exceeds unity (Figs. 2 and SM4) is related to the impact of regional stratification on core dynamics and the geomagnetic field. Indeed, some results from seismology^37^ and mineral physics^38^ favor stable stratification at the top of the core, whereas inferences from the geomagnetic field^39^ and its secular variation^40^ provide evidence against a thick stable layer below the CMB. These contradicting results may be reconciled with partial stratification accommodated by CMB heat flux heterogeneity^19,20^. Our dynamo models highlight a new dynamical aspect of partial stratification. According to our results, partial stratification driven by CMB heat flux heterogeneity (Fig. 4) may suppress regional small-scale contributions to the geomagnetic field which in turn leads to the stabilization of the dipole compared to cases with smaller $q^*$ (Figs. 2 and SM4) and smaller CMB area under subadiabatic conditions.

Further increase beyond $$q^*=2$$, which has not been explored in this study, might lead to failed dynamos. Olson and Christensen^13^ reported for models with two single harmonic outer boundary heat flux patterns ($$Y_2^2$$ and $$Y_2^0$$) that when $$q^*>1$$ the dynamos fail. Likewise, for a particularly localized heat flux pattern (in the context of the past dynamo of Mars), above a critical heterogeneity amplitude cessation of the dynamos was found^41^. For a $$Y_1^0$$ pattern (again relevant to the past Martian dynamo), Yan et al.^42^ found that the dynamo failure depends on the convection style. Furthermore, the dynamo may resurrect for much larger $$q^*$$^42^. Overall, in numerical dynamos with single harmonic outer boundary heat flux patterns the critical $$q^*$$ value for dynamo failure may depend on the internal control parameters as well as the convection style (T. Frisson and N. Schaeffer, personal communication). Our study shows that when considering the multi harmonic tomographic CMB heat flux, self-consistent convection-driven dynamos are obtained until at least $$q^*=2$$, in agreement with previous studies^22,34^.

The results presented here may depend on our choice of convection style. The thermal and chemical buoyancy fluxes in the core are debated. Thermal convection depends on the total and adiabatic CMB heat fluxes, both of which are highly uncertain. In particular, the adiabatic heat flux depends on the thermal conductivity of the liquid core, a material property for which estimates are yet to converge (compare e.g.^38,43^). Taking into account possible ranges for these CMB heat fluxes, both thermal superadiabatic and subadiabatic scenarios are possible^44^. Chemical convection depends on the inner core growth rate, which is also uncertain. Cormier et al.^45^ favor thermochemical core convection with dominant chemical convection, although different combinations of CMB total and adiabatic heat fluxes as well as inner core growth rate, all within their estimated ranges, may give anything from purely chemical convection to even dominant thermal convection! Here we naively set zero buoyancy sources/sinks, corresponding to thermochemical convection with equal thermal and chemical contributions. In a future study, it is worth testing the validity of our results for different partitionings of thermal and chemical buoyancy fluxes. Finally, a systematic parameter study is required to confirm our results for the dependence of the dynamo regime transitions on the amplitude of the CMB heat flux heterogeneity, both the transition from non-reversing to reversing dynamos as well as the transition from reversing to failed dynamos.

## Methods

### Numerical dynamo models

Numerical dynamo simulations are self-consistent solutions to the full set of magnetohydrodynamic (MHD) equations: conservation of momentum, electromagnetic induction, conservation of heat (or co-density), incompressibility and non-existent magnetic monopoles (e.g.^46^). The Boussinesq approximation is applied, and gravity varies linearly with radial distance. These equations in non-dimensional form are respectively (e.g.^46,47^):

1 $$\begin{aligned}{} & {} E \left( \frac{\partial {\textbf {u}}}{ \partial t} + {\textbf {u}} \cdot \nabla {\textbf {u}} - \nabla ^2{\textbf {u}} \right) +2\hat{z}\times {\textbf {u}}+\nabla P = Ra^*\frac{{\textbf {r}}}{r_o}C+\frac{1}{Pm}(\nabla \times {\textbf {B}}) \times {\textbf {B}} \textrm{,} \end{aligned}$$

2 $$\begin{aligned}{} & {} \frac{\partial {\textbf {B}}}{ \partial t} = \nabla \times ({\textbf {u}} \times {\textbf {B}}) + \frac{1}{Pm}\nabla ^2 {\textbf {B}}\textrm{,} \end{aligned}$$

3 $$\begin{aligned}{} & {} \frac{\partial C}{ \partial t} + {\textbf {u}}\cdot \nabla C = \frac{1}{Pr}\nabla ^2C\textrm{,} \end{aligned}$$

4 $$\begin{aligned}{} & {} \nabla \cdot {\textbf {u}} = 0\textrm{,} \end{aligned}$$

5 $$\begin{aligned}{} & {} \nabla \cdot {\textbf {B}} = 0\textrm{,} \end{aligned}$$

where **u** is the fluid velocity, *t* time, $\hat{z}$ the direction of the axis of rotation, *P* the pressure, **r** the position vector, $r_o$ the outer boundary radius, *C* the co-density and **B** the magnetic field. The co-density is given by $C=\alpha T+\beta \xi$ where *T* is temperature, $\xi$ light elements concentration and $\alpha$ and $\beta$ their respective expansivities. Equations (1)–(3) contain four (internal) control parameters. The Ekman number represents the ratio of viscous to Coriolis forces:

6 $$\begin{aligned} E=\frac{\nu }{\Omega D^2}. \end{aligned}$$

The heat flux based Rayleigh number represents the convection vigor vs. retarding forces:

7 $$\begin{aligned} Ra= \frac{\alpha g_0 q_0 D^4}{\kappa \nu k}. \end{aligned}$$

The Prandtl number and the magnetic Prandtl number are ratios of diffusivities:

8 $$\begin{aligned}{} & {} Pr=\frac{\nu }{\kappa }, \end{aligned}$$

9 $$\begin{aligned}{} & {} Pm=\frac{\nu }{\eta }. \end{aligned}$$

Note that the modified Rayleigh number $Ra^*$ in (1) is related to the classical Rayleigh number *Ra* in (7) by $Ra^*=Ra E/Pr$. In (6)–(9) $\Omega$ is the rotation rate, $\nu$ the kinematic viscosity, *D* the shell thickness, $g_0$ the gravitational acceleration at the outer boundary, $q_0$ the mean outer boundary heat flux, *k* the thermal conductivity, $\kappa$ the thermal diffusivity and $\eta$ the magnetic diffusivity.

In all dynamo simulations we imposed rigid and electrically insulating conditions at both boundaries. The spherical shell has an Earth-like inner to outer core radii ratio of 0.35. No volumetric co-density source or sink was assigned, i.e. thermal bouyancy sources (primarily secular cooling) and chemical bouyancy sinks (light elements release from the freezing of the inner core) are assumed to balance each other, corresponding to thermochemical convection^15^. On the outer boundary of the simulations a heat flux pattern was imposed based on a tomographic model of seismic shear wave velocity anomalies at the lowermost mantle^28^ truncated at spherical harmonic degree and order 6. The amplitude of the imposed heat flux heterogeneity is quantified by (e.g.^13^)

10 $$\begin{aligned} q^*=\frac{q_{max}-q_{min}}{2q_0}\textrm{,} \end{aligned}$$

where $q_{max}$ and $q_{min}$ are the maximum and minimum heat flux respectively. For the inner boundary fixed co-density was imposed.

Several main outputs characterize the convection in the models. The magnetic Reynolds number, which measures the ratio of magnetic field advection to diffusion, is defined by

11 $$\begin{aligned} Rm = \frac{UD}{\eta } \end{aligned}$$

where *U* is the rms velocity in the shell volume. In our dynamo models $Rm\sim 1500$ (Table SM1), somewhat larger yet on the same order of magnitude as the estimate for Earth’s core (e.g.^48^). The Rossby number is a conventional measure of the ratio of inertial to Coriolis forces:

12 $$\begin{aligned} Ro = \frac{U}{\Omega D} \end{aligned}\rm{.}$$

The local Rossby number^4^ accounts for the actual flow length-scale:

13 $$\begin{aligned} Ro_\ell = \frac{Ro\bar{\ell }_u}{\pi } \end{aligned}$$

where $\ell _u$ is the characteristic wave-number of the flow obtained from the time-averaged kinetic energy spectrum:

14 $${\begin{aligned} \bar{\ell }_u = \frac{\sum \ell \langle {\textbf {u}}_\ell \cdot {\textbf {u}}_\ell \rangle }{2E_{kin}} \end{aligned}}\rm{.}$$

Other outputs characterize the modeled magnetic fields, in particular the dipole. The relative dipole field strength on the outer boundary is defined by

15 $$\begin{aligned} f_{dip}=\left( \frac{ \frac{4}{3} \left( (g_1^0)^2+(g_1^1)^2+(h_1^1)^2\right) }{\sum _{\ell =1 }^{12}\sum _{m=0}^{\ell } \frac{(\ell +1)^2}{2\ell +1} \left( (g_\ell ^m)^2+(h_\ell ^m)^2\right) }\right) ^{1/2} \end{aligned}$$

where $\ell$ and *m* are the spherical harmonics degree and order respectively. In addition we calculate the time-average dipole tilt. In the reversing dynamos we fold the tilt angle to the northern hemisphere so that the resulting quantity $<\theta _{dip}>$ represents the average distance from the geographic pole ($<>$ denotes time averaging). Because a model in which a reversal has not been observed is not guaranteed to be non-reversing, $<\theta _{dip}>$ serves as a practical measure of reversibility for finite simulation times (which is always the case). In Table SM1 the $<\theta _{dip}>$ values are given for all models. Note that in the non-reversing models ($q^*=0-0.75$) $<\theta _{dip}>$ is below 10$^\circ$, whereas in the reversing models ($q^*=0.85-2$) $<\theta _{dip}>$ is $\sim$20$^\circ$ and above.

In the dynamo models output, time is given in units of viscous diffusion times $\tau _\nu =D^2/\nu$. The magnetic diffusion time $\tau _\eta =D^2/\eta$ is simply related to the viscous diffusion time by $\tau _\eta =Pm\tau _\nu$. In the context of magnetic field reversals, it is common practice to express the results in terms of the dipole diffusion time^49^ $\tau _{dip}=\frac{r_o^2}{\pi ^2 \eta }$. In terms of the viscous diffusion time,

16 $$\begin{aligned} \tau _{dip}=Pm\left( \frac{r_o}{\pi D} \right) ^2 \tau _\nu \end{aligned}\rm{.}$$

For Earth’s core $\tau _{dip} \sim$ 38 kyrs.

### Regional measures of boundary control

Heterogeneous boundary control on the convection in the shell and the resulting magnetic field are quantified by focusing on specific regions at the top of the shell. Large outer boundary heat flux regions are defined by a heat flux anomaly larger than half the maximum, i.e. $\delta q > \frac{1}{2} \delta q_{max}$. To quantify regional triggering of reversals (relevant for moderate $q^*<1$), we measure the time-average ratio between the RMS of the azimuthal component of the inertial force at $r/r_o=0.95$ outside/inside regions where $\delta q > \frac{1}{2} \delta q_{max}$ which we denote as $<I_{out}/I_{q^+}>$, with

17 $$\begin{aligned}<I_{q^+}>= & {} <\sqrt{ \frac{\int _{q^+} I_\phi ^2 dS}{\int _{q^+} dS}}> \end{aligned}$$

18 $$\begin{aligned}<I_{q_{out}}>= & {} <\sqrt{\frac{\int _{q_{out}} I_\phi ^2 dS}{\int _{q_{out}} dS}}>\end{aligned}$$

where the surface increment is $dS=r^2 \sin \theta d\phi d\theta$. The integrations in (17) are over the large heat flux regions where $\delta q > \frac{1}{2} \delta q_{max}$, whereas the integrations in (18) are over the remaining CMB surface.

In contrast, to quantify regional suppression of reversals (relevant for larger $$q^*>1)$$, we measure the time-average ratio between the RMS radial velocity at $$r/r_o=0.95$$ inside/outside regions where $$\delta q < \frac{1}{2} \delta q_{min}$$ which we denote as $$<u_r{_{q^-}}/u_r{_{out}}>$$, with

19 $$\begin{aligned}<u_r{_{q^-}}>= & {} <\sqrt{ \frac{\int _{q^-} u_r^2 dS}{\int _{q^-} dS}}>\end{aligned}$$

20 $$\begin{aligned}<u_r{_{q_{out}}}>= & {} <\sqrt{ \frac{\int _{q_{out}} u_r^2 dS}{\int _{q_{out}} dS}}>\end{aligned}$$

The integrations in (19) are over the low heat flux regions where $\delta q < \frac{1}{2} \delta q_{min}$, while the integrations in (20) are over the remaining CMB surface.

## Supplementary Information

Supplementary Information 1.

Supplementary Information 2.

## Data Availability

Data will be provided upon request.
