# Supplementary material for: Regionally-triggered geomagnetic reversals
Source: Sci Rep. 2024 Apr 26;14:9639. doi: 10.1038/s41598-024-59849-z (PMC11577040; doi:10.1038/s41598-024-59849-z)
Supplement: Supplementary file 1 — Supplementary Information 1. [file 41598_2024_59849_MOESM1_ESM.pdf]

# Regionally-triggered geomagnetic reversals

Filipe Terra-Nova[1,*] and Hagay Amit[1]

## Supplementary Material

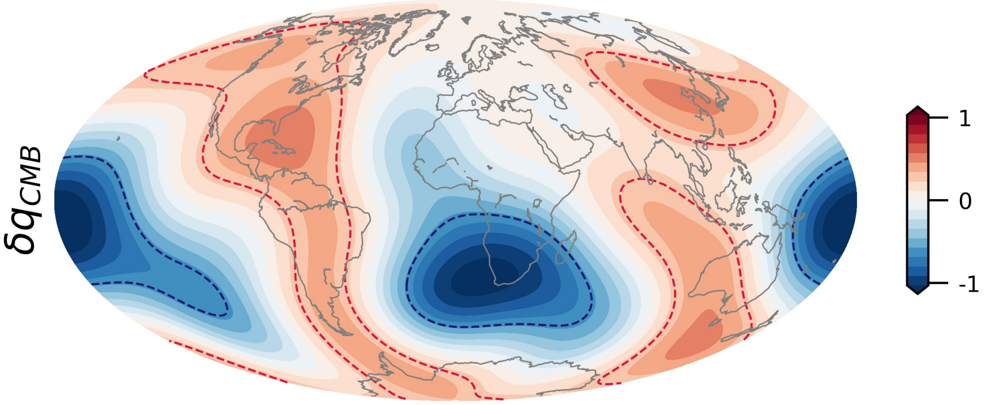


**Figure SM1:** Non-dimensional heterogeneous heat flux anomalies imposed on the outer boundary of the dynamo models based on a tomographic model of seismic shear wave velocity anomalies at the lowermost mantle [1] truncated at spherical harmonic degree and order 6. Dashed red and blue contours denote half maximum and half minimum values.

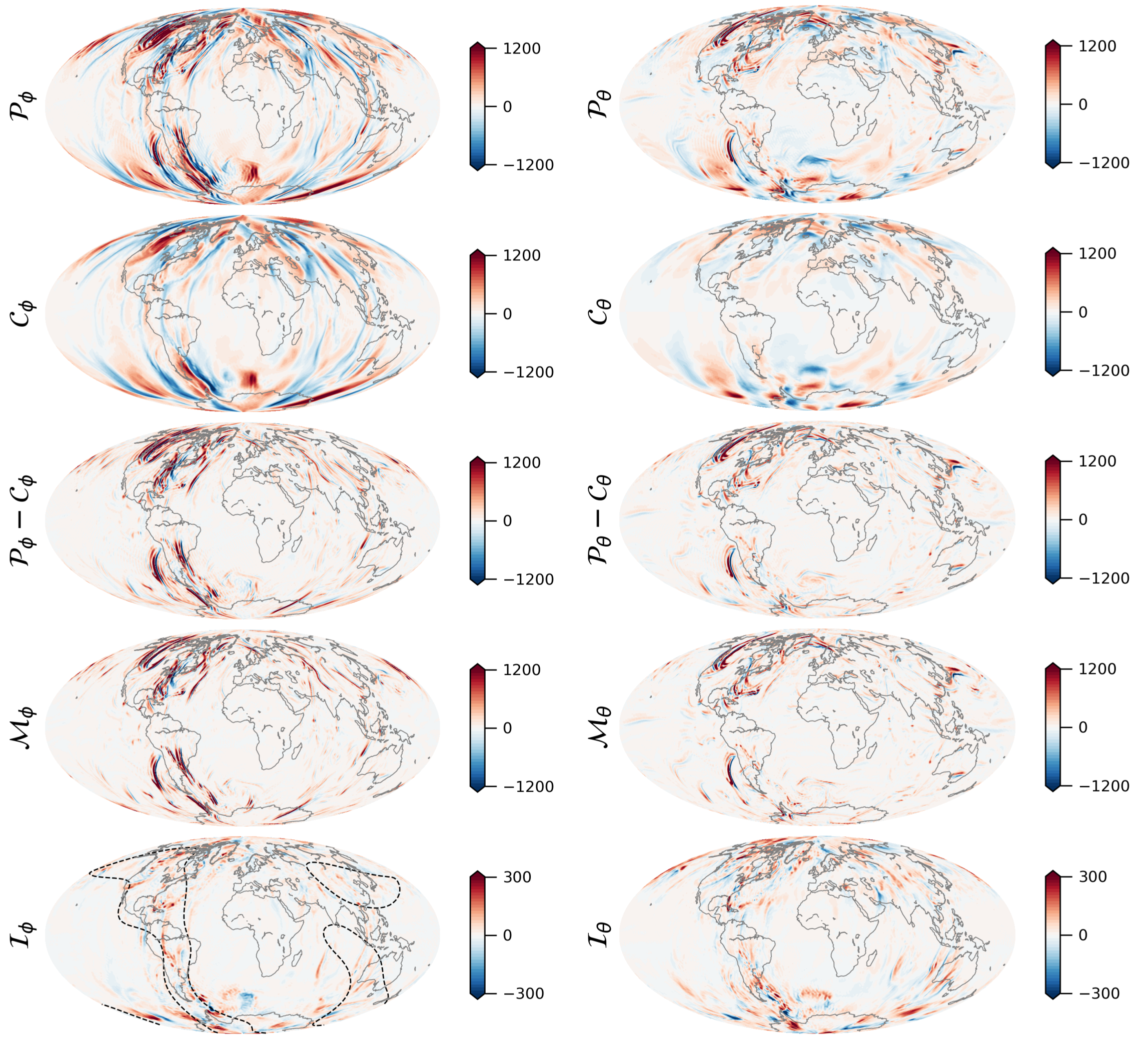


**Figure SM2:** Azimuthal (subscript $\phi$, left) and meridional (subscript $\theta$, right) components of the Pressure gradient, Coriolis, ageostrophic Coriolis, Lorentz and inertia forces ($\mathcal{P}$, $\mathcal{C}$, $\mathcal{P} - \mathcal{C}$, $\mathcal{M}$ and $\mathcal{I}$) at the top of the shell ($r/r_o = 0.95$) for a snapshot of the model with $q^* = 1.0$. Note the different scale for the inertia term.

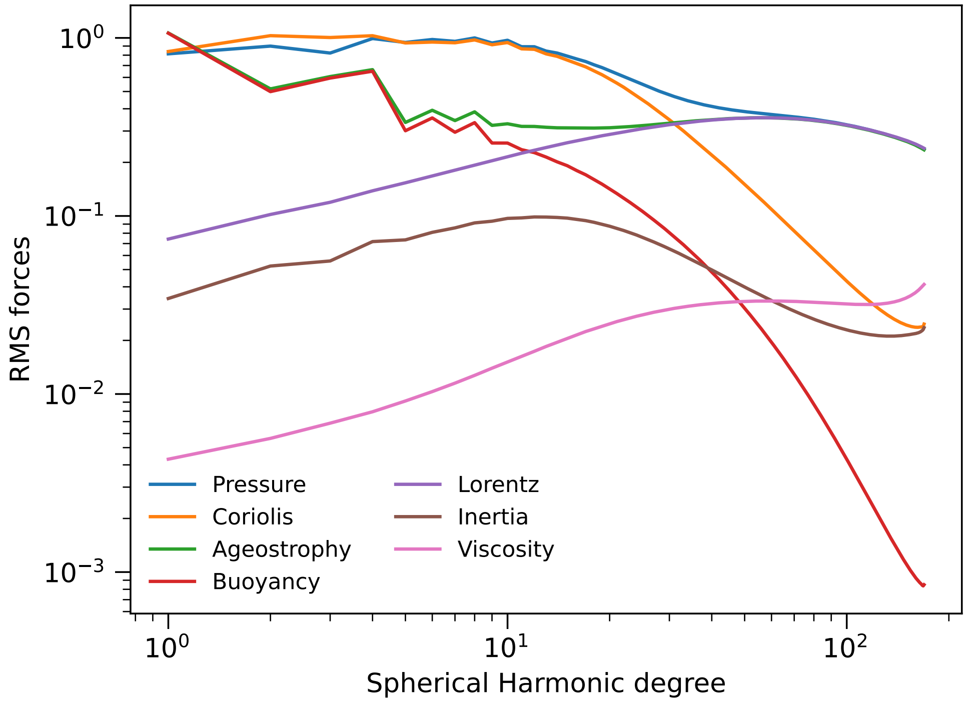


**Figure SM3:** RMS of the forces in the volume of the shell excluding the viscous boundary layers (top and bottom of the shell) as in [2] for the time average of the model with $q^* = 1.0$. The RMS forces are normalized by the peak of the pressure gradient force.

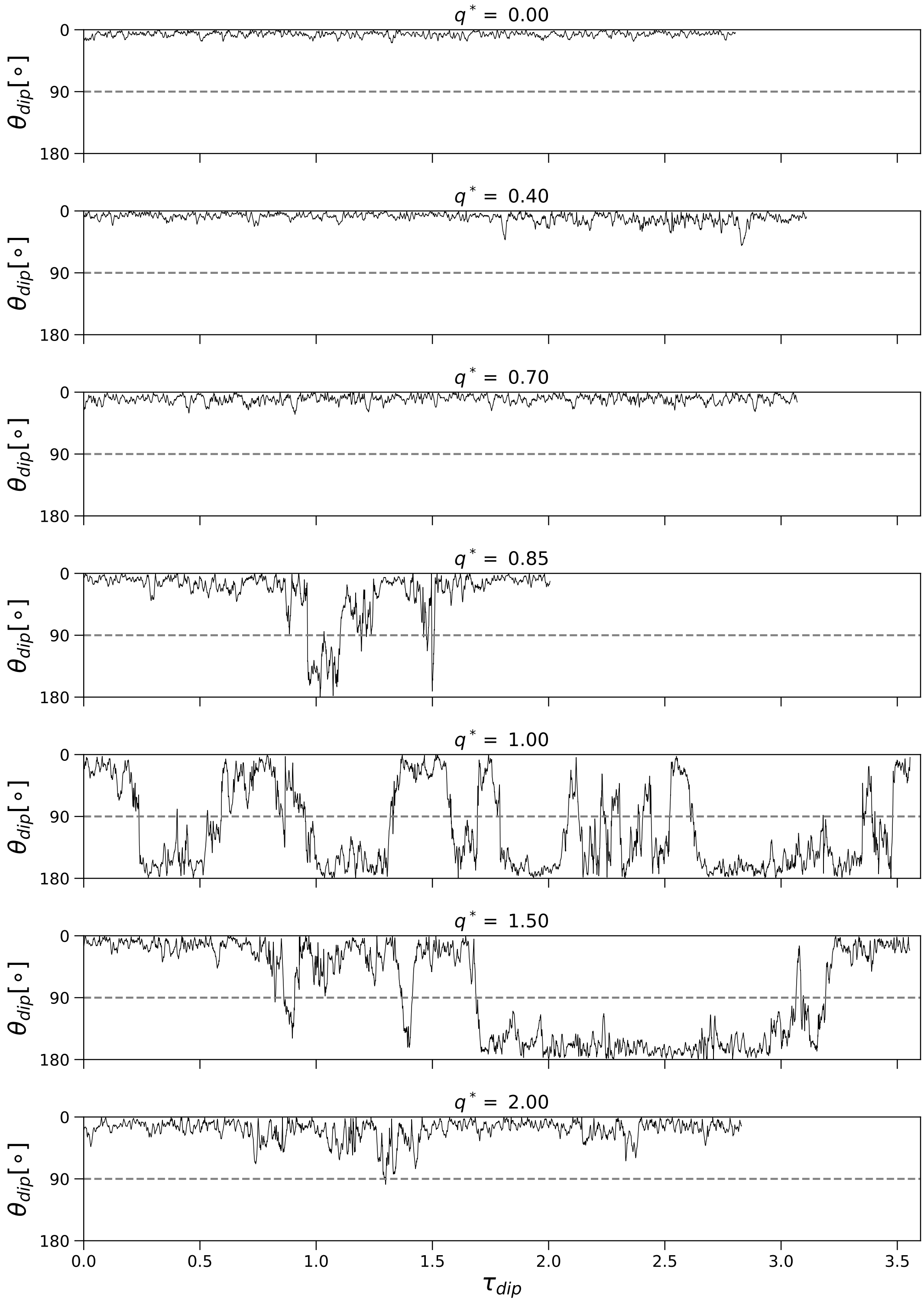


**Figure SM4:** The distance of the dipole axis from the north geographic pole vs. time in units of dipole diffusion times for the numerical dynamo models. In all cases $E = 1 \times 10^{-4}$, $Ra = 4 \times 10^7$, $Pr = 1$ and $Pm = 8$. Increasing $q^*$ values are indicated above each timeserie.

**Table 1:** Mean values of some outputs of the numerical dynamo simulations.

| $q^*$ | $Rm$ | $f_{dip}$ | $Ro_\ell$ | $E_{mag}/E_{kin}$ | $\mathcal{I}_{out}/\mathcal{I}_{q^+}$ | $u_{rq^-}/u_{rout}$ | $\theta_{dip}$ |
|---|---|---|---|---|---|---|---|
| 0.00 | 1406.69 | 0.55 | 9.085e-02 | 3.10 | 1.038 | 0.979 | 6.52±3.45 |
| 0.40 | 1462.24 | 0.46 | 9.500e-02 | 2.53 | 0.960 | 0.713 | 9.39±6.19 |
| 0.70 | 1485.14 | 0.42 | 9.574e-02 | 2.28 | 0.896 | 0.526 | 10.30±5.77 |
| 0.85 | 1534.29 | 0.36 | 9.920e-02 | 1.88 | 0.890 | 0.466 | 18.36±16.16 |
| 1.00 | 1592.57 | 0.20 | 1.032e-01 | 1.44 | 0.921 | 0.391 | 31.94±22.66 |
| 1.50 | 1590.74 | 0.24 | 1.009e-01 | 1.55 | 0.845 | 0.310 | 24.23±18.44 |
| 2.00 | 1536.80 | 0.34 | 9.097e-02 | 2.48 | 0.775 | 0.293 | 16.84±12.24 |

In all models the Ekman number (6) is $E = 1\times10^{-4}$, the Rayleigh number (7) is $Ra = 4\times 10^7$, the Prandtl number (8) is $Pr = 1$ and the magnetic Prandtl number (9) is $Pm = 8$. $q^*$ is the amplitude of the imposed heat flux heterogeneity (10), $Rm$ the magnetic Reynolds number (11), $Ro_\ell$ the local Rossby number (13) and $f_{dip}$ a measure of magnetic field dipole strength (15). $E_{mag}$ and $E_{kin}$ are the magnetic and kinetic energies in the entire shell, respectively. $\mathcal{I}_{out}/\mathcal{I}_{q^+}$ denotes the concentration of inertial forces inside regions where $\delta q > \frac{1}{2}\delta q_{max}$ (17-18). $u_{rq^-}/u_{rout}$ denotes the suppression of radial velocity field inside regions where $\delta q < \frac{1}{2}\delta q_{min}$ (19-20). $\theta_{dip}$ is the time-average distance of the dipole axis from the north geographic pole (see Methods) and its standard deviation is also given.

**Movie:** Radial magnetic field on the outer boundary truncated at spherical harmonic degree and order 14 from the dynamo model with $q^* = 1.5$ during a reversal.

# References

[1] Masters, G., Laske, G., Bolton, H. & Dziewonski, A. The relative behavior of shear velocity, bulk sound velocity, and compressional velocity in the mantle: Implications for chemical and thermal structure. In, *Earth's Deep Interior: Mineral Physics and Tomogra-*

*phy From the Atomic to the Global Scale*, Karato, S., Forte, A., Liebermann, R., Masters, G. & Stixrude, L. (eds), Vol. 117 (AGU monograph, Washington D.C., 2000).

[2] Aubert, J., Gastine, T. & Fournier, A. Spherical convective dynamos in the rapidly rotating asymptotic regime. *J. Fluid. Mech.* **813**, 558–593 (2017).
